# Calycosin attenuates renal ischemia/reperfusion injury by suppressing NF-κB mediated inflammation *via* PPARγ/EGR1 pathway

**DOI:** 10.3389/fphar.2022.970616

**Published:** 2022-10-07

**Authors:** Ningxin Zhang, Chen Guan, Zengying Liu, Chenyu Li, Chengyu Yang, Lingyu Xu, Meng Niu, Long Zhao, Bin Zhou, Lin Che, Yanfei Wang, Yan Xu

**Affiliations:** Department of Nephrology, The Affiliated Hospital of Qingdao University, Qingdao, China

**Keywords:** calycosin, acute kidney injury, ischemia/reperfusion injury, peroxisome proliferator-activated receptor γ, early growth response 1

## Abstract

Renal ischemia reperfusion injury (IRI) is a leading and common cause of acute kidney injury (AKI), and inflammation is a critical factor in ischemic AKI progression. Calycosin (CAL), a major active component of *Radix astragali*, has been reported to have anti-inflammatory effect in multiple organs. However, whether CAL can alleviate renal IRI and its mechanism remain uncertain. In the present study, a renal IRI model is established by bilateral renal pedicles occlusion for 35 min in male C57BL/6 mice, and the effect of CAL on renal IRI is measured by serum creatinine and pathohistological assay. Hypoxia/reoxygenation (H/R) stimulated human renal tubular epithelial cells HK-2 were applied to explore the regulatory mechanisms of CAL. Luciferase reporter assay and molecular docking were applied to identify the CAL’s target protein and pathway. In the mice with renal IRI, CAL dose dependently alleviated the renal injury and decreased nuclear factor kappa B (NF-κB) mediated inflammatory response. Bioinformatics analysis and experiments showed that early growth response 1 (EGR1) increased in mice with renal IRI and promoted NF-κB mediated inflammatory processes, and CAL dose-dependably reduced EGR1. Through JASPAR database and luciferase reporter assay, peroxisome proliferator-activated receptor γ (PPARγ) was predicted to be a transcription factor of EGR1 and repressed the expression of EGR1 in renal tubular epithelial cells. CAL could increase PPARγ in a dose dependent manner in mice with renal IRI and molecular docking predicted CAL could bind stably to PPARγ. In HK-2 cells after H/R, CAL increased PPARγ, decreased EGR1, and inhibited NF-κB mediated inflammatory response. However, PPARγ knockdown by siRNA transfection abrogated the anti-inflammation therapeutic effect of CAL. CAL produced a protective effect on renal IRI by attenuating NF-κB mediated inflammatory response *via* PPARγ/EGR1 pathway.

## Introduction

Acute kidney injury (AKI) is defined as a rapid decrease in kidney function with increased serum creatinine levels and/or decreased urine output. AKI occurs in approximately 10%–15% of hospitalized patients, which is increasingly recognized as a risk factor for chronic kidney disease (CKD) (Ronco et al., 2019; Rashid et al., 2022). However, there is no effective prevention and treatment for AKI (Zhou et al., 2020; Li et al., 2021). Renal ischemia reperfusion injury (IRI) is a leading and common cause of AKI, and delayed recovery of IRI contributes to chronic kidney disease (CKD) and even end-stage kidney disease (Pefanis et al., 2019; Husain-Syed et al., 2021). Therefore, it is important to explore effective intervention for ischemic AKI.

Previous studies indicated that renal IRI is closely associated with the oxidative stress and inflammatory response (Andrade-Oliveira et al., 2019; Sato et al., 2020), which is especially relevant to renal tubular epithelial cells (TECs) injury (Tammaro et al., 2020) that contributing to the overall renal damage. Nuclear factor kappa B (NF-κB)-mediated pro-inflammatory response plays a critical role in AKI (Song et al., 2019). The underlying mechanism study showed that NF-κB activation triggers the release of inflammatory cytokines such as interleukin-1beta (IL-1β), interleukin-6 (IL-6) and tumor necrosis factor alpha (TNF-α) that exacerbating kidney injury (Place and Kanneganti., 2018). Therefore, targeting NF-κB pathway is a promising anti-inflammation treatment which necessary to prevent or treat ischemic AKI.

Early growth response protein 1 (EGR1), a zinc-finger transcription factor of the immediate early gene family, has been reported to promote the release of pro-inflammatory mediators by activating NF-κB and exacerbate renal damage (Ho et al., 2016). However, the effect of EGR1 on inflammation in renal IRI has not been clarified. Peroxisome proliferator-activated receptor γ (PPARγ) is an important transcriptional factor, which belongs to superfamily of nuclear receptors. Previous studies have shown a relationship between PPARγ and EGR1, among which activation PPARγ can inhibit EGR1 to suppress inflammation responses (Li and Xia, 2020). However, the regulatory relationship between EGR1 and PPARγ and their role in inflammatory response in renal IRI remain unclear.

Natural products have been considered an alternative therapy for the treatment of various renal diseases including AKI and CKD (Geng et al., 2020; Meng et al., 2020; Fang et al., 2021; Shao et al., 2021; He et al., 2022). Calycosin, an isoflavonoid phytoestrogen isolated from *Radix astragali*, has various pharmacological activities including anti-cancer, anti-inflammatory, anti-oxidant and neuroprotective effects (Deng et al., 2021; Wang et al., 2022). Recent research has focus on the anti-inflammatory effect of CAL, such as suppressing neuroinflammation *via* blocking NF-κB pathway and NLRP3 inflammasome (Chen et al., 2020). However, whether CAL has a protective effect on renal IRI and its mechanism remains elusive. In this study, we investigate CAL effect on ischemic AKI and underlying mechanisms.

## Materials and methods

### Drug

CAL (CAS: 20575-57-9, purity ≥98.0%, MedChem Express, NJ, United States) was suspended in 10% dimethyl sulfoxide (DMSO) and 90% corn oil for mice administration. For cell experiment, CAL was dissolved in DMSO to make a 1 mg/ml stock solution.

### Animals protocol

**FIGURE 1:**
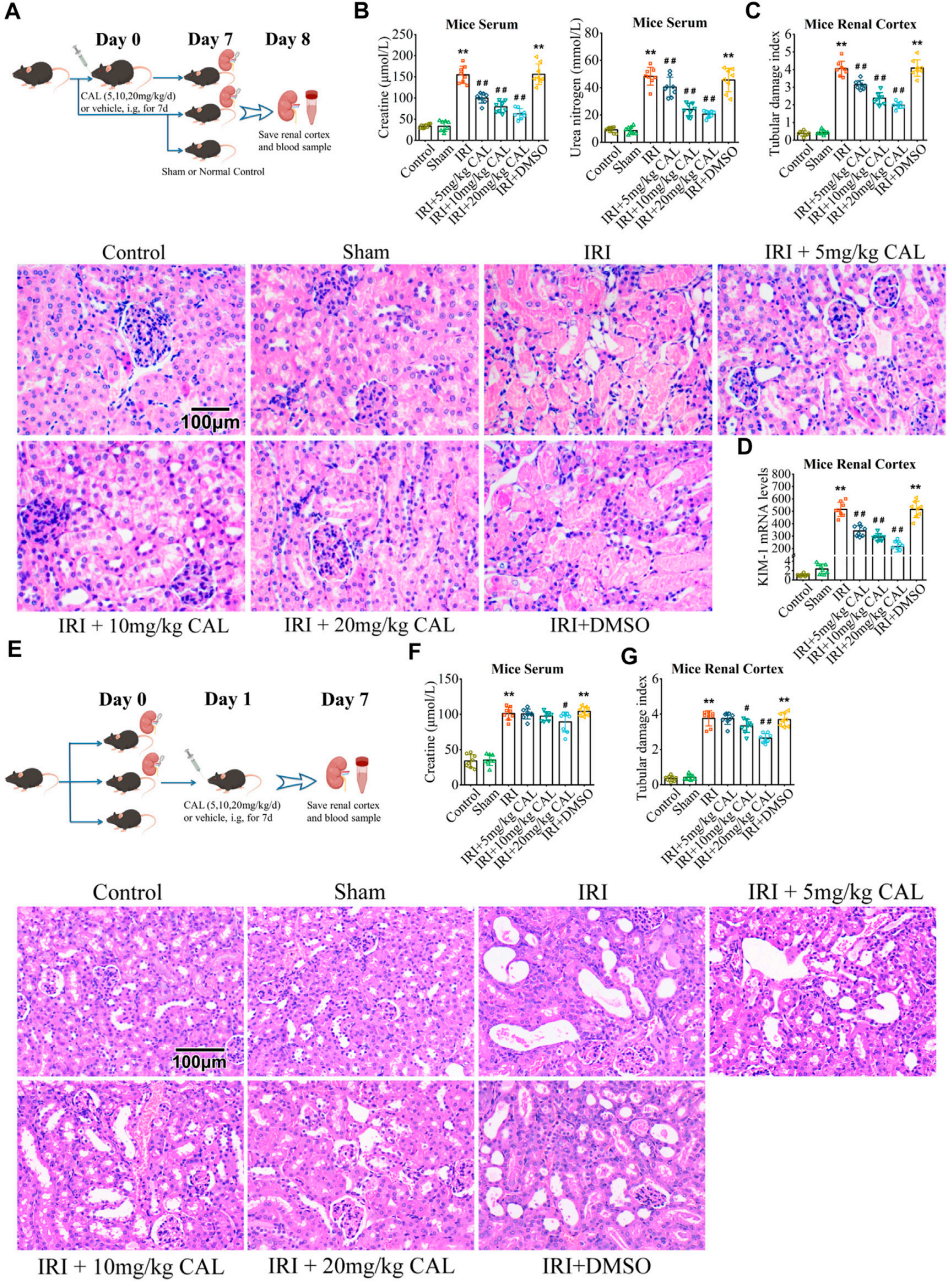
CAL protected the kidney from IRI. **(A)** The pattern diagram of the animal research design for CAL pre-treatment. **(B)** SCr and BUN levels in kidney samples of different groups. **(C)** Renal tubular damage index and H and E staining images of kidney tissue samples in different groups. Scale bar, 100 µm. **(D)** KIM-1 mRNA level in kidney samples of different groups. ^**^ *p* < 0.01 vs. sham group. ^##^ *p* < 0.01 vs. IRI group. *n* = 8 per group. **(E)** The pattern diagram of the animal research design for CAL post-surgery administration. **(F)** SCr level in kidney samples of different groups. **(G)** Renal tubular damage index and H and E staining images of kidney tissue samples in different groups. Scale bar, 100 µm. ^**^ *p* < 0.01 vs. sham group. ^#^ *p* < 0.05, ^##^ *p* < 0.01 vs. IRI group. *n* = 8 per group. The data are presented as the mean ± SD.

C57BL/6 male mice (6–8weeks) were purchased from Pengyue Laboratory Animal Co., Ltd. (Jinan, China). Mice were randomly allocated into seven groups (eight per group): control, sham, IRI, IRI + 5 mg/kg CAL, IRI +10 mg/kg CAL, IRI +20 mg/kg CAL, IRI + DMSO. CAL was intragastrically given daily for 7 days before IRI modeling (Figure 1A). To establish the renal IRI model, mice were anesthetized with intraperitoneal pentobarbital and subjected to bilateral renal artery occlusion for 35 min followed by reperfusion 24 h. The kidneys were observed until the color turned bright red which confirmed reperfusion. The sham operation was identical to the treatment operation, except for renal pedicle clamping. To explore the therapeutic effect of post-surgery CAL administration on IRI, some mice was subjected to bilateral renal artery occlusion for 35 min followed by reperfusion 24 h, and then treated with CAL for 7 days. Some mice were treated with CAL intragastrically without IRI operation. Blood samples were obtained from the orbital sinus and renal cortex tissue samples were stored at −80°C until use. Animal care and experimental procedures were approved by the Laboratory Animal Welfare Ethics Committee at Qingdao University (ethics number: 202105C5730202106036).

### Renal function assessment

The renal function was assessed by measuring serum creatinine (SCr) and blood urea nitrogen (BUN) levels. The samples were evaluated using commercially available kits (Nanjing Jiancheng Bioengineering Institute, Nanjing, China) according to the manufacturer’s recommended protocol.

### Histopathological studies

Kidney tissue samples were collected after IRI and fixed in 10% formaldehyde overnight. The samples were embedded in paraffin, cut into 4-µm-thick sections. And then stained with hematoxylin and eosin (H & E) (Solarbio, Beijing, China) is performed. Renal tubular damage was scored according to the extent of foamy degeneration and the detachment of tubular cells on a semiquantitative scale.

### Cell lines and cell treatment

Human renal tubular epithelial cells HK-2 were purchased from the Procell Life Science and Technology (Wuhan, China). HK-2 cells were cultured in DMEM/F-12 medium (Biological Industries, Kibbutz Beit Haemek, Israel) mixed with 10% fetal bovine serum (Gibco, NY, United States) and 100 × penicillin-streptomycin solution (Solarbio, Beijing, China) and incubated in a 37°C humidified incubator in an atmosphere of 5% CO_2_.

CAL solutions at different concentrations were prepared for incubation with HK-2 cells for 24 h, and equal volumes of DMSO were added to the vehicle groups. For Hypoxia/reoxygenation (H/R) treatment, cells were plated to 80% confluence, and exposed to 24 h of hypoxia (5% CO_2_, 1% O_2_, and 94% N_2_) followed by 3 h of reoxygenation. The experimental groups are as follows: control, H/R, H/R + 8 μM CAL, H/R + 16 μM CAL, H/R + 32 μMCAL, H/R + DMSO.

### Cell viability detection

Cell Counting Kit-8 (CCK-8, MedChem Express, NJ, United States) was used for cell viability assessment. HK-2 cells were seeded in 96-well plates followed by incubation with graded concentrations of CAL for 24 h. Then 10 μl CCK-8 was added into each well and incubated for 2 h at 37°C. A microplate reader (Thermo Fisher Scientific Inc., MA, United States) was used for the measurement of absorbance at 450 nm.

### Western blot

The mice kidney tissues and cells were lysed in RIPA lysis buffer (Elabscience Biotechnology, Wuhan, China). Protein samples were subjected to SDS-PAGE, followed by transfer to polyvinylidene fluoride membranes (PVDF, Millipore, MA, United States). After blocked, membranes were incubated at 4°C overnight with primary antibodies as follows: anti-phospho-IκBα (1:1000, Cell Signaling Technology, MA, United States), anti-IκBα (1:1000, Cell Signaling Technology, MA, United States),anti-phospho-NF-κB p65 (1:1000, Cell Signaling Technology, MA, United States),anti-NF-κB p65 (1:1000, Cell Signaling Technology, MA, United States), anti-β-actin (1:1000, Proteintech, IL, United States), anti-EGR1 (1:2000, Cell Signaling Technology, MA, United States), anti-PPARγ (1:1000, Cell Signaling Technology, MA, United States). The secondary antibody, goat anti-rabbit IgG-HRP (1:5000, Elabscience Biotechnology, Wuhan, China), was incubated at room temperature for 1 h and detected using Excellent Chemiluminescent Substrate (ECL) detection kit (Elabscience Biotechnology, Wuhan, China). The bands were subjected to gray scale analysis using ImageJ (version 1.8.0) software.

### Enzyme linked immunosorbent assay

The levels of IL-1β, IL-6, and TNF-α of mice serum and cell culture supernatants were determined by an ELISA system (Elabscience Biotechnology, Wuhan, China) according to the manufacturer’s protocols.

### Quantitative real-time PCR

Total RNA isolation was performed using Trizol (Invitrogen, MA, United States). cDNA was synthesized using a HiScript RT SuperMix (Vazyme Biotech, Nanjing, China). qRT-PCR was performed using ChamQ Universal SYBR qPCR Master Mix (Vazyme Biotech, Nanjing, China), and the data were normalized to the expression of β-actin. The primers used are shown in Supplementary Table S1.

### Immunohistochemical

EGR1 and PPARγ immunohistochemical were performed as described previously (Singh et al., 2019). Primary antibody: anti-EGR1 antibody (1:500, Proteintech, IL, United States), anti-PPARγ antibody (1:200, Elabscience Biotechnology, Wuhan, China). EGR1 and PPARγ area ratio was analyzed using ImageJ (version 1.8.0) software.

### Bioinformatics

Raw microarray data of GSE52004 (Affymetrix Mouse Gene 1.0 ST Array platform) were downloaded from GEO Datasets (https://www.ncbi.nlm.nih.gov/gds). The microarray data were contributed by Liu et al. (2014). The raw data were preprocessed using a robust multi-array averaging algorithm in *R* (version 4.0.4) software. The *Limma* package was loaded for differential expression analysis. The adjusted *p* value (adj. *P*) was calculated with the Benjamini and Hochberg method. DEGs were identified with absolute log_2_FC > 3 and adj. *p* < 0.05.

PPI information was acquired from the STRING database (http://www.string-db.org/). Medium confidence (0.4) was chosen as the minimum required interaction score to screen the interactions among DEGs. PPI network visualization was conducted by Cytoscape (version 3.9.1) software. Hub genes were selected by Maximal Clique Centrality methods with a criterion of score >100 (Chin et al., 2014).

Molecular docking was carried out by Auto Dock 4.2.6 (The Scripps Research Institute). Briefly, proteins were selected as rigid receptor molecules, while CAL was selected as the ligand for protein docking. PDB files of proteins were downloaded from the RCSB Protein Data Bank (http://www.rcsb.org/pdb/home/home.do). The SDF file for CAL was downloaded from the NCBI PubChem Compound database (https://www.ncbi.nlm.nih.gov/pccompound). After the receptor molecule and ligand files were prepared, a grid box was set for the movement and rotation of the ligand. Finally, docking was accomplished *via* the AutoDock4 program. The visual simulation was conducted using the PyMOL 2.5 (DeLano Scientific LLC, CA, United States).

### *In vitro* transfection experiment

HK-2 cells were plated into 6-well plates. When grown to 60% confluence, the cells were transfected with siRNA against target gene or control siRNA (GenePharm, Shanghai, China) using Lipofectamine 3,000 (Invitrogen, MA, United States). After 24 h, knockdown of target gene expression was validated by qRT-PCR analysis and cultures with more than 80% reduction in target gene mRNA expression comparing to the control cultures were included in further experiments.

### Luciferase reporter assay

The promoter sequence of EGR1 was cloned into the pGL3 vector (Promega, WI, United States) upstream of the luciferase sequence (pGL3-EGR1). 293T cells were seeded into 24-well plates. When grown to 60% confluence, 293T cells were co-transfected with the 0.5 μg PPARγ over-expression plasmid (PPARγ-OE), 0.5 μg pGL3-EGR1 and 0.02 μg Renilla plasmid (pRL-TK) using X-tremegene HP reagent (Roche, Basel, Switzerland). And CAL (8 μM) were prepared for incubation with cells. After 48 h, the firefly and Renilla luciferase activities were measured with a Dual-Luciferase Reporter Assay System (Promega, WI, United States). The firefly luciferase activity was normalized to Renilla luciferase activity.

### Statistical analyses

Values are expressed as the mean ± SD from at least three experiments. Statistical significance was analyzed by ANOVA followed by the Bonferroni *post hoc* test in SPSS 25.0 statistical software. A value of *p* < 0.05 was considered statistically significant.

## Results

### Calycosin improved kidney function and reduced renal inflammation responses

**FIGURE 2:**
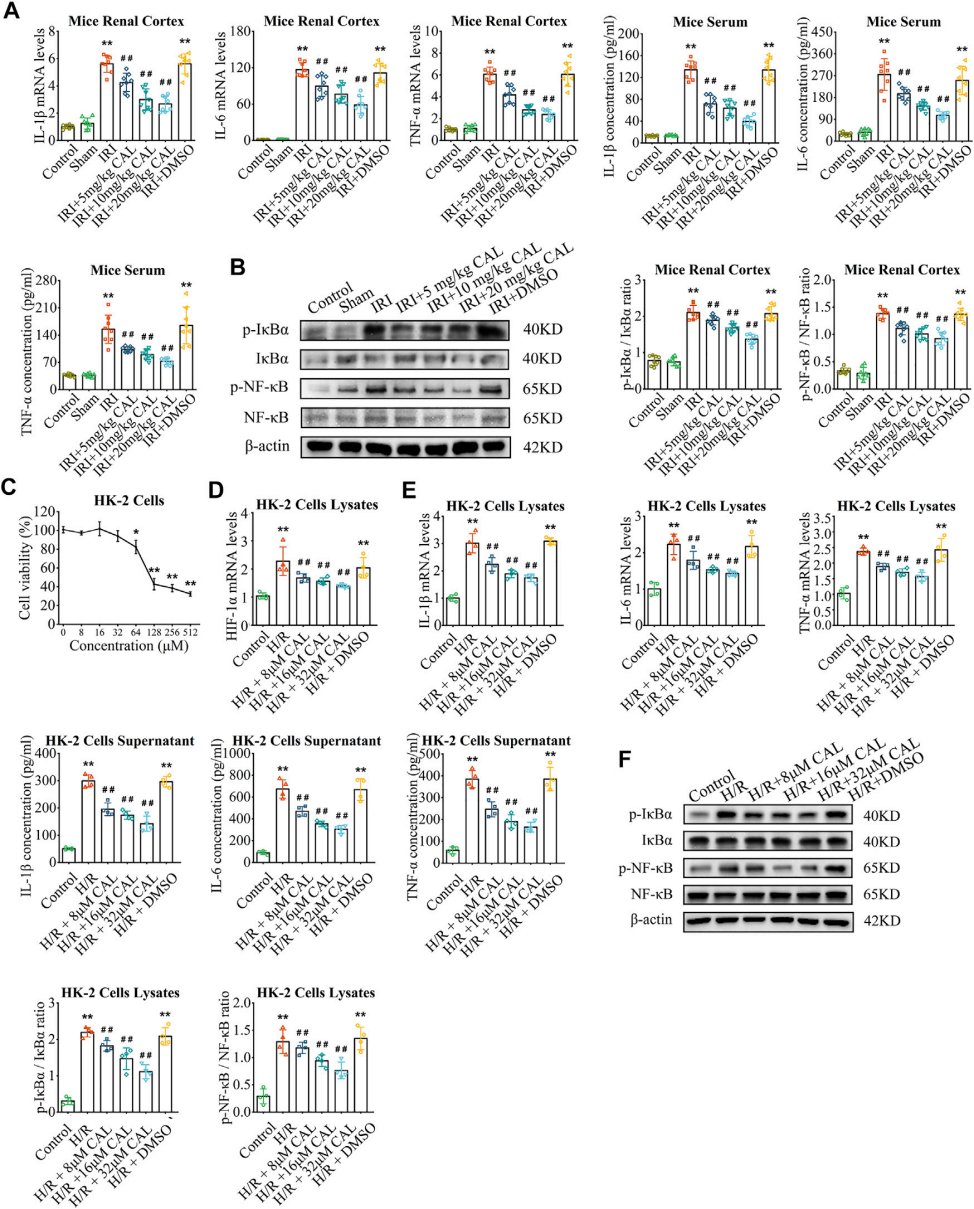
CAL ameliorated inflammation response in mice after IRI and H/R-induced HK-2 cells. **(A)** IL-1β, IL-6, and TNF-α mRNA levels in kidney samples and IL-1β, IL-6, and TNF-α concentrations in mice serum of different groups. **(B)** The protein levels of p-NF-κB/NF-κB and p-IκBα/IκBα in kidney samples of different groups. ^**^ *p* < 0.01 vs. sham group. ^##^ *p* < 0.01 vs. IRI group. *n* = 8 per group. **(C)** Results of CCK-8 testing of different concentrations of CAL on HK-2 cells. ^*^ *p* < 0.05, ^**^ *p* < 0.01 vs. control cells. **(D)** The HIF-1α mRNA level in different cell groups. **(E)** IL-1β, IL-6, and TNF-α mRNA levels in cell lysates and IL-1β, IL-6, and TNF-α concentrations in supernatant of different groups. **(F)** The protein levels of p-NF-κB/NF-κB and p-IκBα/IκBα in cell lysates of different groups. ^**^ *p* < 0.01 vs. control group. ^##^ *p* < 0.01 vs. H/R group. *n* = 4 per group. The data are presented as the mean ± SD.

After IRI 24 h, mice exhibited elevated SCr and BUN levels, while CAL pretreatment significantly reduced both in a dose-dependent manner (Figure 1B). The IRI kidneys showed more renal tubular injury with high kidney pathological injury score, but 20 mg/kg CAL treatment reduced approximately half of the score (Figure 1C). Furthermore, CAL significantly decreased the level of kidney injury molecule-1 (KIM-1) in renal cortex compared with IRI group (Figure 1D). The effect of post-surgery CAL administration on IRI was explored (Figure 1E), and the results showed that CAL treatment after IRI preserved more kidney function and alleviated the renal pathological change (Figures 1F,G). To investigate the anti-inflammatory effects of CAL on IRI-induced AKI, the mRNA levels and concentrations of inflammatory cytokines, such as IL-1β, IL-6, and TNF-α, were detected. As shown in Figure 2A, the inflammatory cytokines significantly increased after IRI, whereas CAL pretreatment markedly inhibited them. Meanwhile, we found that IRI significantly increased NF-κB activation and IκBα degradation. However, treatment of CAL dose-dependently inhibited IRI-induced NF-κB activation (Figure 2B).

*Invitro*, the toxicity of CAL was negligible at concentrations of 32 μM or less for 24 h (Figure 2C). H/R induced HK-2 cells model was applied to mimic renal IRI, and H/R treatment led to a significant increase in hypoxia-inducible factor 1α (HIF-1α) expression in HK-2 cells, whereas CAL incubation reduced the levels of HIF-1α in a dose-dependent manner (Figure 2D). Likewise, the inflammatory cytokines were induced by treatment with H/R in HK-2 cells and were decreased by incubation with CAL (Figure 2E). And CAL inhibited H/R induced NF-κB activation in HK-2 cells (Figure 2F). These results suggested that CAL alleviates renal function injury and inhibits excessive inflammation in TECs after H/R.

### Early growth response 1 involved in the anti-inflammatory effect of calycosin

**FIGURE 3:**
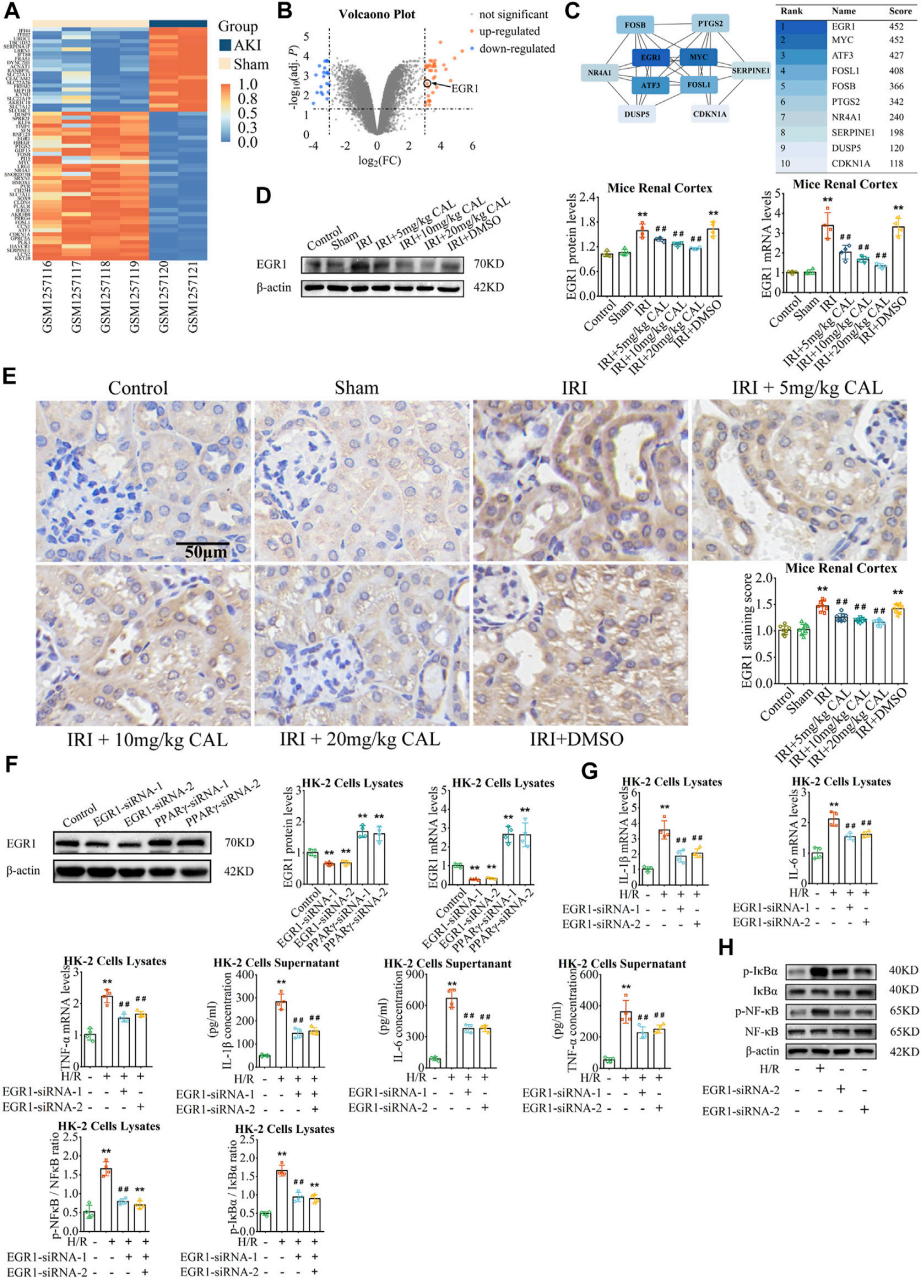
EGR1 involved in the anti-inflammatory effect of CAL. **(A)** Heatmap of GSE52004. Absolute log_2_FC > 3 with adj. *p* < 0.05 was considered the threshold of DEGs (orange: high expression; blue: low expression). **(B)** Volcanic plot of the DEGs (orange: up-regulated; blue: down-regulated). **(C)** PPI network of top 10 hub genes selected by the Maximal Clique Centrality method *via* Cytoscape. **(D)** The EGR1 protein and mRNA levels in kidney samples of different groups. **(E)** Immunohistochemistry showed the expression of EGR1 of kidney tissue samples in different groups. Scale bar, 50 µm. ^**^ *p* < 0.01 vs. sham group. ^##^ *p* < 0.01 vs. IRI group. *n* = 8 per group. **(F)** The protein and mRNA expression of EGR1 in HK-2 cells transfected with EGR1 siRNA or PPARγ siRNA. ^**^ *p* < 0.01 vs. control group **(G)** IL-1β, IL-6, and TNF-α mRNA levels in cell lysates and IL-1β, IL-6, and TNF-α concentrations in cell supernatant of different groups. **(H)** The protein levels of p-NF-κB/NF-κB and p-IκBα/IκBα in cell lysates of different groups. ^**^ *p* < 0.01 compared with control group. ^##^ *p* < 0.01 compared with the H/R group. *n* = 4 per group. The data are presented as the mean ± SD.

We identified 58 DEGs in the kidneys of IRI mice (Figures 3A,B; Supplementary Table S2), and ten hub genes were selected (Figure 3C). Among these hub genes, early growth response 1 (EGR1) had the highest score and upregulated markedly in IRI (adj. *p* < 0.01). *In vivo*, both protein and mRNA levels of EGR1 are significantly increased after IRI (Figure 3D). The EGR1 mainly expressed in TECs in the renal cortex, which increased significantly after IRI (Figure 3E), while CAL pretreatment showed a significant dose-dependent decrease of EGR1 expression in TECs. *In vitro*, EGR1 siRNA was transferred into HK-2 cells to knock down EGR1 (Figure 3F). Compared to the H/R group, the expression of inflammatory cytokines and NF-κB activation were suppressed after EGR1 knock down (Figures 3G,H). Taken together, EGR1 was upregulated after IRI, and involved in the inflammatory response of TECs.

### Peroxisome proliferator-activated receptor γ targeted and inhibited early growth response 1

**FIGURE 4:**
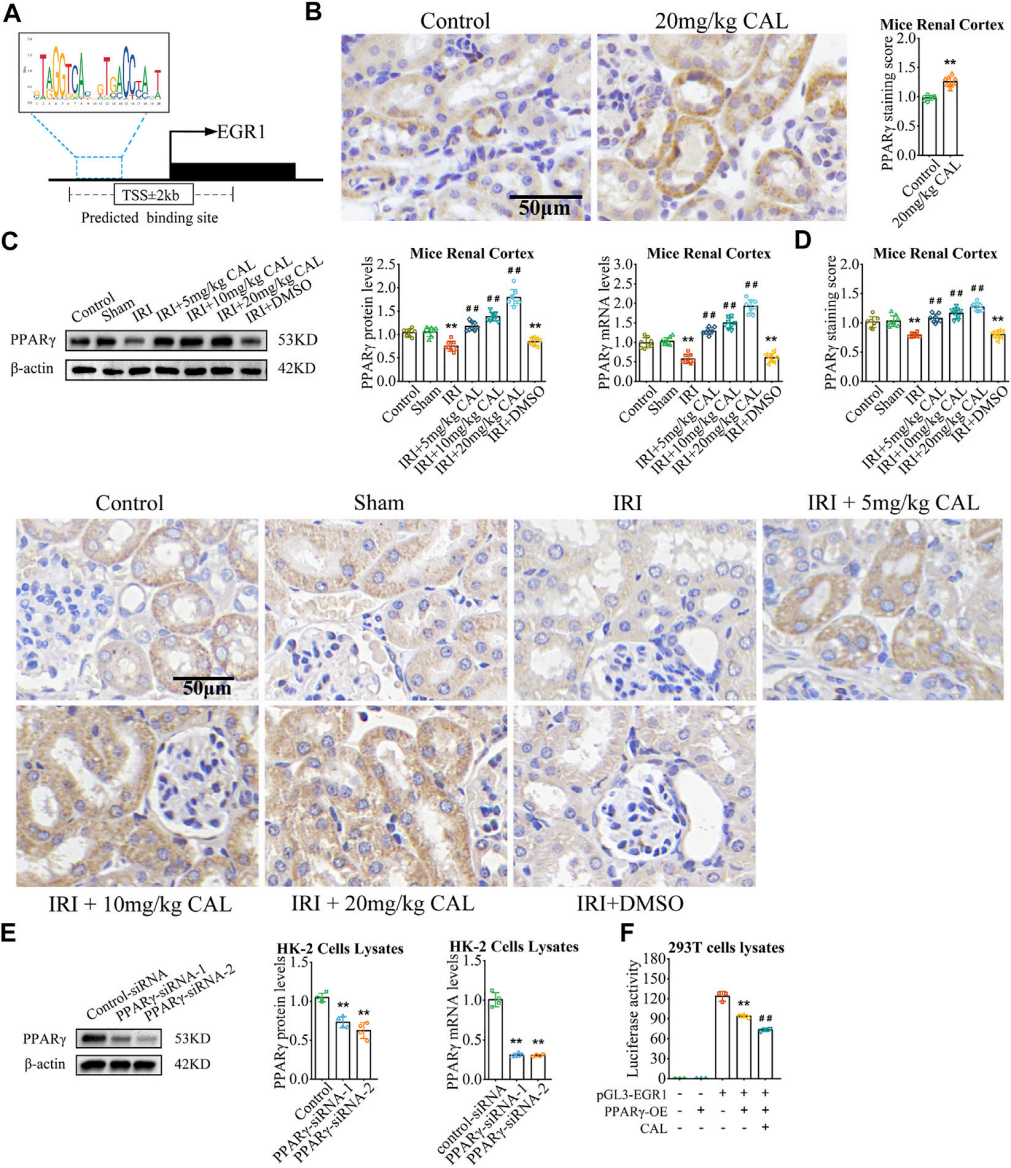
PPARγ targeted and inhibited EGR1. **(A)** The binding sites of PPARγ between EGR1 promoter region predicted by JASPAR website. **(B)** Immunohistochemistry showed the expression level PPARγ of kidney tissue samples in normal control group and 20 mg/kg CAL group. Scale bar, 50 µm. ^**^ *p* < 0.01 vs. control group. *n* = 3 per group. **(C)** The PPARγ mRNA and protein levels in kidney samples of different groups. **(D)** Immunohistochemistry showed the expression level PPARγ of kidney tissue samples in different groups. Scale bar, 50 µm. ^**^ *p* < 0.01 vs. sham group. ^##^ *p* < 0.01 vs. IRI group. *n* = 8 per group. **(E)** The mRNA and protein levels of PPARγ in HK-2 cells transfected with PPARγ siRNA or control siRNA. ^**^ *p* < 0.01 vs. control siRNA group. *n* = 4 per group. **(F)** Luciferase reporter assay showed the effect of CAL and PPARγ on the activities of EGR1 promoters. ^**^ *p* < 0.01 vs. pGL3-EGR1 without PPARγ-OE group. ^##^ *p* < 0.01 vs. pGL3-EGR1 with PPARγ-OE group. *n* = 3 per group. The data are presented as the mean ± SD.

In order to study the upstream of EGR1, we explored the transcriptional factor that might bind to the promoter region of EGR1. As the result, PPARγ is one of the factors that targeting the EGR1 with a relative score = 0.766 (Figure 4A). *In vivo*, PPARγ expressed in renal cortex and was significantly reduced after IRI compared with the sham group, but CAL promote PPARγ expression (Figures 4B,C). *In vitro*, PPARγ siRNA significantly increased the expression of EGR1 (Figures 3F, 4D), indicating that PPARγinhibitEGR1 in tubular cells. Moreover, luciferase reporter assay confirmed that PPARγ interact with EGR1 promoter, as EGR1 increased after EGR1 promoter plasmid transfection but decreased after PPARγ overexpression (Figure 4E). Meanwhile, the repressive effect of PPARγ on the EGR1 promoter was more obvious upon CAL incubation (Figure 4E). Taken together, these results indicated that PPARγ inhibited EGR1 expression and represses the activity of the EGR1 promoter.

In order to study the upstream of EGR1, we explored the transcriptional factor that might bind to the promoter region of EGR1. As the result, PPARγ is one of the factors that targeting the EGR1 with a relative score = 0.766 (Figure 4A). *In vivo*, CAL administration improved PPARγ expression in renal cortex (Figure 4B). Meanwhile, PPARγ was significantly reduced after IRI compared with the sham group, but CAL promoted PPARγ expression (Figures 4C,D). *In vitro*, PPARγ siRNA significantly increased the expression of EGR1 (Figures 3F, 4E), indicating that PPARγ inhibit EGR1 in tubular cells. Moreover, luciferase reporter assay confirmed that PPARγ interact with EGR1 promoter, as EGR1 increased after EGR1 promoter plasmid transfection but decreased after PPARγ overexpression (Figure 4F). Meanwhile, the repressive effect of PPARγ on the EGR1 promoter was more obvious upon CAL incubation (Figure 4F). Taken together, these results indicated that PPARγ inhibited EGR1 expression and represses the activity of the EGR1 promoter.

### Calycosin inhibited inflammation *via* peroxisome proliferator-activated receptor γ/early growth response 1

**FIGURE 5:**
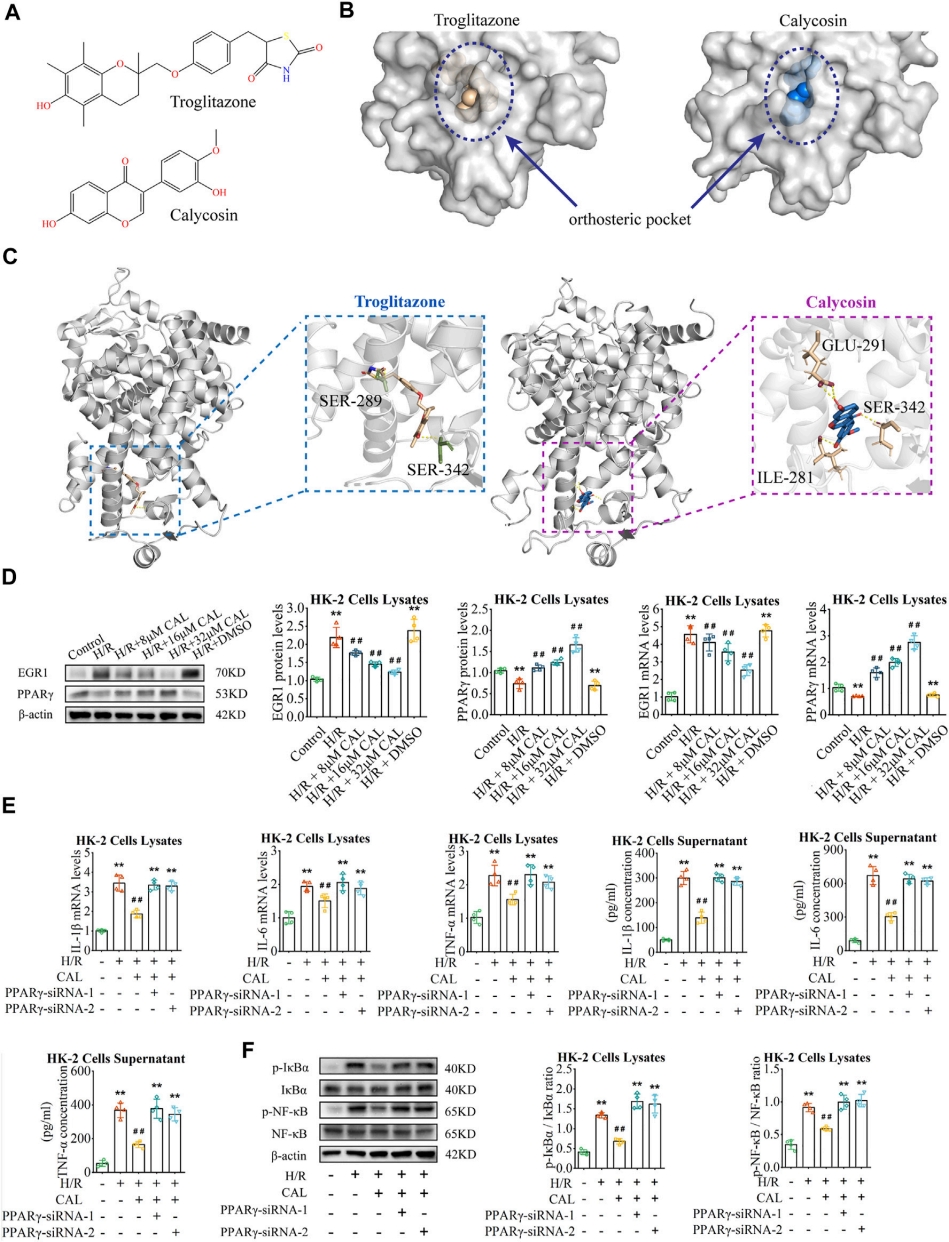
CAL inhibited inflammation *via* PPARγ/EGR1. **(A)** Chemical structure of troglitazone and CAL. **(B)** Structure of PPARγ revealed troglitazone and CAL is located in the orthosteric pocket of PPARγ. Troglitazone and CAL are shown as the spheres structure, and PPARγ is shown as the surfaces structure. **(C)** Troglitazone and CAL (ligand) bound to PPARγ (receptor) *via* hydrogen bonds. Ligand are shown as the stick structure, and PPARγ is shown as the cartoon structure. Hydrogen bonds are shown as dotted lines (yellow). ILE, isoleucine; SER, serine; GLU, glutamate. **(D)** The PPARγ and EGR1 mRNA and protein levels in different cell groups. **(E)** IL-1β, IL-6, and TNF-α mRNA levels in cell lysates and IL-1β, IL-6, and TNF-α concentrations in supernatant of different groups. **(F)** The protein levels of p-NF-κB/NF-κB and p-IκBα/IκBα in cell lysates of different groups. ^**^ *p* < 0.01 vs. control group. ^##^ *p* < 0.01 vs. H/R group. *n* = 4 per group. The data are presented as the mean ± SD.

As a ligand, CAL could be embedded into the orthosteric pocket of PPARγ with a binding energy of -6.46 kcal/mol, which is exactly the binding pocket of thiazolidinedione agonists represented by troglitazone (Figures 5A,B; Supplementary Table S3). Interestingly, CAL formed hydrogen bonds with the residues Ser342, Ile281, and Glu291, in the ligand binding cavity of PPARγ, whereas troglitazone with the residues Ser342 and Ser289 (Figure 5C). This suggests that CAL probably activate PPARγ through a canonical thiazolidinedione ligand-binding cavity in a similar manner with troglitazone.

*In vitro*, compared with H/R induced cells, PPARγ increased and EGR1 decreased considerably in the CAL incubation groups (Figure 5D). However, the protective effect of CAL on H/R induced inflammation was absent when PPARγ knockdown, with increase of inflammatory cytokines and NF-κB activation (Figures 5E, F). These results indicated the PPARγ plays a role in CAL protection against H/R induced cells. Thus, we suggested that CAL might ameliorate renal IRI and inhibit inflammation *via* PPARγ/EGR1.

## Discussion

The present study provided a novel approach and mechanism for the treatment of ischemic AKI. The main findings are as follows: 1) CAL alleviated renal injury and suppressed excessive inflammation response in renal IRI. 2) The protective effect of CAL was accessed by targeting the PPARγ/EGR1 pathway.

Oxidative stress and inflammation play a vital role in the pathophysiology of various diseases, such as senescence, cardiovascular disease, cancer and AKI (Li et al., 2020; Ikeda et al., 2021; Radovanovic et al., 2021; Zhang. et al., 2021; Wen et al., 2022). The activation of NF-κB signaling pathway has been identified as a major contributor of inflammation in the injured kidneys (Zhu et al., 2021). Consistent with this, in the present study, activation of NF-κB and inflammatory cytokines such as IL-1β, IL-6, and TNF-α are significantly increased in IRI mice, while CAL has reported to effectively inhibit inflammation by inhibiting NF-κB in various organ models (Zhang et al., 2019; Zhu et al., 2021). Of note, CAL treatment showed a good anti-inflammatory effect with the decrease of proinflammatory cytokines and inhibition of NF-κB activation in kidney after IRI.

To investigate the potential mechanisms of CAL, we applied differential gene analysis and PPI network analysis, and the results showed that EGR1 as a hub gene is significantly elevated in ischemic AKI. EGR1, a zinc-finger transcription factor of the immediate early gene family, is known to modulate inflammation responses in various tissues (Pritchard et al., 2010; Yan et al., 2021). Particularly, Ho et al. (2016) demonstrated that EGR1 aggravates tubulointerstitial nephritis by promotes NF-κB/NLRP3 in tubular cells. Consistent with this, we found that EGR1 expression in TECs was elevated after IRI stimulation and H/R stimulation, and proinflammatory cytokines were reduced and NF-κB activation was reduced when EGR1 knockdown, suggesting that EGR1 is involved in promoting the inflammatory response in TECs. In the IRI mice model, CAL dose-dependently decreased EGR1 in TECs after IRI, suggesting that the anti-inflammatory effect of CAL is related to EGR1.

PPARγ is a transcriptional factor belonging to nuclear receptors (NRs) superfamily (Wang et al., 2016; Gao et al., 2021). Previous studies have found that the activation of PPARγ inhibits EGR1 to suppress inflammation responses in brain and lung (Li et al., 2020; Yan et al., 2021), but the specific mechanism remains unclear. In the present study, EGR1 was upregulated significantly in HK-2 cells after PPARγ knockdown. Furthermore, luciferase reporter assay suggested that PPARγ repressed the activity of the EGR1 promoter, which provided a possibility to explain the regulatory relationship between PPAR and EGR1. In IRI stimulated mice and H/R induced HK-2 cells, the expression of PPARγ decreased, which are consistent with the previous findings (Singh et al., 2019; Wei et al., 2019). One of the possible reasons is that pro-inflammatory stimulation causes a decrease in the expression of PPARγ (Korbecki et al., 2019). A regulatory feedback loop between the PPARγ and TLR4 pathways may partly explain this phenomenon. When stimulated by LPS or other stimuli, TLR4 acts as an initial signal transduction, triggering the activation of pro-inflammatory signaling pathways, and preventing anti-inflammatory activity by negatively regulating PPARγ (Cao et al., 2018). Activation of PPARγ can negatively regulate the expression of TLR4 gene and attenuate the activation of downstream inflammatory signaling pathways (Mateu et al., 2015). Besides, prostaglandin (PG)H_2_ is specifically catalyzed to PGE_2_ by mPGES-1 under inflammatory conditions. The pro-inflammatory PEG_2_ can also inhibit the expression of PPARγ through pathways involving PI3K and Akt signaling (Kapoor et al., 2007; García-Alonso et al., 2013).

Extensive studies have demonstrated that many natural compounds are PPARγ agonist (Bernardo et al., 2021; Ji et al., 2021; Zhang et al. (2021), Zhang et al., 2020; Zhu et al., 2020). CAL as an isoflavonoid is reported to be an effective PPARγ agonist (Shen et al., 2006). This phenomenon was supported by molecular docking evidence, showing that CAL bind PPARγ stably by forming hydrogen bonds with residues Ser342, Ile281, and Glu291 in the orthosteric pocket of PPARγ. The binding patterns of agonists of PPARγ affect the recruitment of co-activators and co-repressors, playing an important role in modulating target gene expression (Jang et al., 2017). identified that interaction with Ser342 could be essential for transrepression of hypoxia inducible factor-1alpha (HIF-1α) by ligand-activated PPARγ, since Ser342 mutation abolished the transrepression effect of PPARγ on HIF-α by recruiting the co-repressors SMRT (Zhang et al., 2018). The mechanism by which CAL binds to PPARγ affects the expression of downstream target genes is still unclear, but the results of molecular docking provided a potential direction for further research.

Furthermore, although CAL may facilitate the activation PPARγ resulting in inhibiting inflammation, the potential role of CAL regulation of PPARγ expression in this context could not be excluded. We found that CAL has increased the expression of PPARγ in kidney after IRI and tubular cells after H/R. Increased protein abundance of PPARγ may facilitate its nuclear transcriptional activity. We thus speculated that CAL suppression of inflammation could also be partially attributed to upregulation of PPARγ expression. The siRNA-mediated knockdown of PPARγ expression could validate this speculation.

However, there are still some limitations to this study. The effect of CAL on the transcriptional activity of PPARγ and the binding site of PPARγ regulating the EGR1 promoter still require further experiments to explore. In addition, the regulation of inflammation by PPARγ involves multiple signaling pathways, such as transrepressing of NF-κB expression, inhibiting of NF-κB p65 activation, suppressing of AP-1 activation and inhibiting the AP-1 DNA binding activity, increasing SOCS3 expression to disrupt JAK2/STAT3 activation and many more (Pascual et al., 2005; Yu et al., 2008; Wang et al., 2021). In this work, we only explored that CAL inhibits inflammatory responses through the PPARγ/EGR1 pathway, but CAL may also exert anti-inflammatory effects through other signaling pathways, and further studies are needed.

In summary, our study demonstrated that CAL could significantly modulate inflammation by targeting the PPARγ/EGR1 pathway, contributing to the alleviation of renal IRI (Supplementary Figure S1). CAL, as a novel PPARγ activator, may serve as a potential candidate for pharmaceutic prevention and therapy of ischemic AKI. While the current *in vivo* and *in vitro* models supported the effect of CAL to attenuate ischemic AKI, additional studies will be required to determine whether this strategy could also be used in clinical settings.

## Data Availability

The datasets presented in this study can be found in online repositories. The names of the repository/repositories and accession number(s) can be found in the article/Supplementary Material.
