# Supplementary material for: Calycosin attenuates renal ischemia/reperfusion injury by suppressing NF-κB mediated inflammation via PPARγ/EGR1 pathway
Source: Front Pharmacol. 2022 Oct 7;13:970616. doi: 10.3389/fphar.2022.970616 (PMC9585199; doi:10.3389/fphar.2022.970616)
Supplement: Supplementary file 1 [file Table1.DOCX]

Supplementary Material

# Supplementary Figures

#
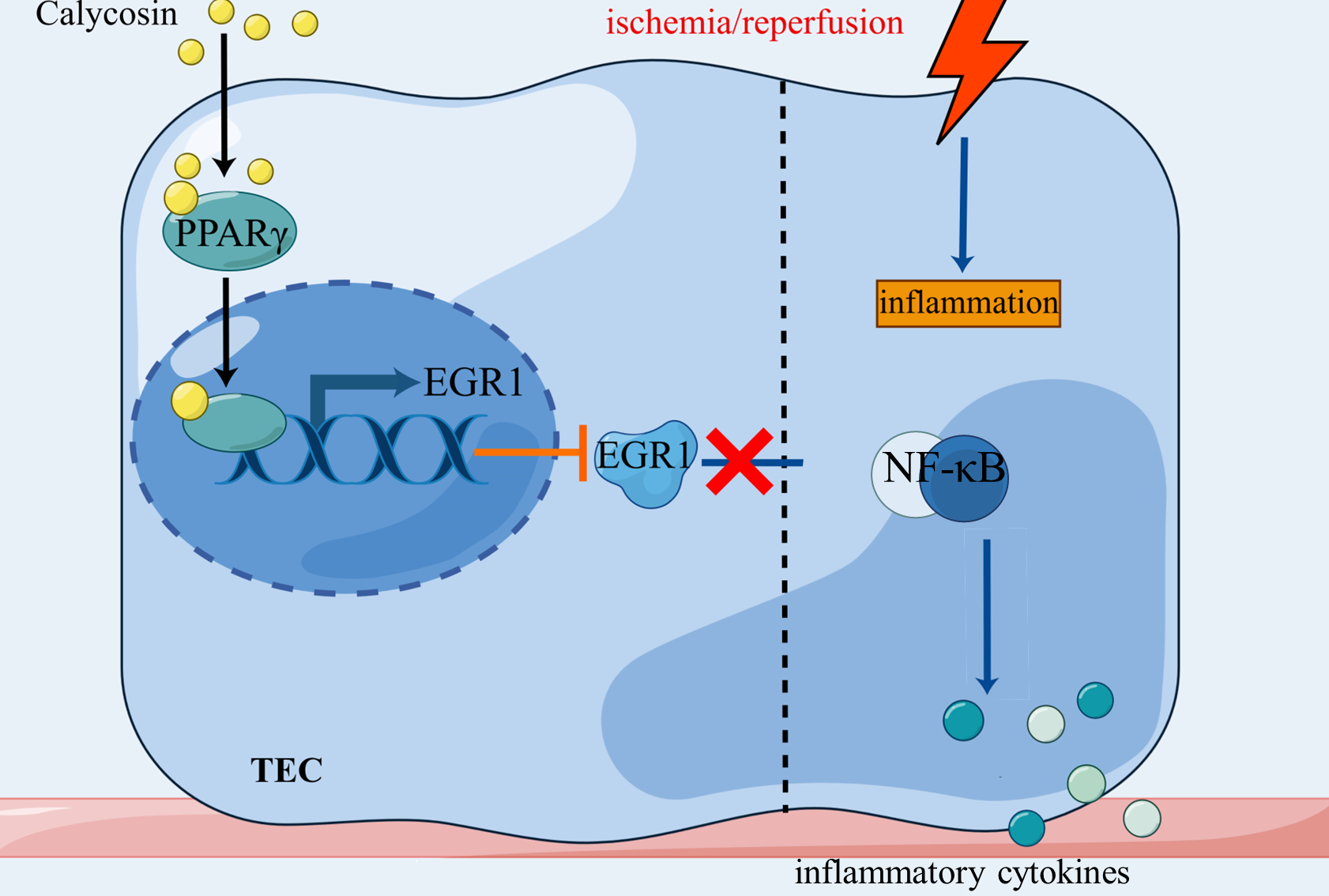


**Supplementary Figure 1.** Schematic diagram of this study. CAL protects renal IRI by targeting PPARγ/EGR1 pathway to alleviate NF-κB mediated inflammatory responses.

# Supplementary Tables

Supplementary Table 1 Primers Used in qRT-PCR.

| **Gene** | **Forward primer** | **Reverse primer** |
| --- | --- | --- |
| KIM-1 (mice) | ACTCCTGCAGACTGGAATGG | CAAAGCTCAGAGAGCCCATC |
| HIF-1α (human) | ACCGCTGAAACGCCAAAG | TCCATCGGAAGGACTAGGTGTCT |
| IL1β (mice) | CTTCTTGGGACTGATGCTGGT | CTCTGTGAAGTCTCCTCTCCG |
| IL1β (human) | TGCCACCTTTTGACAGTGATG | TGATGTGCTGCTGCGAGATT |
| IL6 (mice) | ACCACTCCCAACAGACCTGTCT | CAGATTGTTTTCTGCAAGTGCAT |
| IL6 (human) | TGGCTGAAAAAGATGGATGCT | TCTGCACAGCTCTGGCTTGT |
| TNFα (mice) | ACAAGGCTGCCCCGACTAC | TGGGCTCATACCAGGGTTTG |
| TNFα (human) | TGTAGCCCATGTTGTAGCAAACC | GAGGACCTGGGAGTAGATGAGGTA |
| EGR1 (mice) | GCAGCGGCGGTAATAGCA | CTCCACCATCGCCTTCTCAT |
| EGR1 (human) | AGACCAGTTACCCCAGCCAAA | GTGGGTTGGTCATGCTCACTAG |
| PPARγ (mice) | CATTCTGGCCCACCAACTTC | CAGCTCTTGTGAATGGAATGTCTT |
| PPARγ (human) | TCATGCTTGTGAAGGATGCAA | ATCCCCACTGCAAGGCATT |
| β-actin (mice) | ACGGCCAGGTCATCACTATTG | AGAGGTCTTTACGGATGTCAACGT |
| β-actin (human) | GGGAAATCGTGCGTGACATT | GGAACCGCTCATTGCCAAT |

Supplementary Table 2 Details for group samples extracted from AKI series.

| **GEO series** | **AKI 24h group samples** | **Sham 24h group samples** | **Characters of samples** |
| --- | --- | --- | --- |
| [GSE52004](https://www.ncbi.nlm.nih.gov/geo/query/acc.cgi?acc=GSE52004) | [GSM1257116](https://www.ncbi.nlm.nih.gov/geo/query/acc.cgi?acc=GSM1257116), [GSM1257117](https://www.ncbi.nlm.nih.gov/geo/query/acc.cgi?acc=GSM1257117),  [GSM1257118](https://www.ncbi.nlm.nih.gov/geo/query/acc.cgi?acc=GSM1257118),  [GSM1257119](https://www.ncbi.nlm.nih.gov/geo/query/acc.cgi?acc=GSM1257119) | [GSM1257120](https://www.ncbi.nlm.nih.gov/geo/query/acc.cgi?acc=GSM1257120),  [GSM1257121](https://www.ncbi.nlm.nih.gov/geo/query/acc.cgi?acc=GSM1257121) | C57BL/6 mice |

Supplementary Table 3 Details about the binding between CAL and PPARγ.

| **Protein** | **PDB ID** | **Binding Energy (kcal/mol)** | **Amino Acid** |
| --- | --- | --- | --- |
| PPARγ | 7AWD | -6.46 | ILE-281, GLU-291, SER-342 |

ILE, isoleucine; SER, serine; GLU, glutamate.
